# Cleft Lip and Palate Antenatal Diagnosis: A Swiss University Center Performance Analysis

**DOI:** 10.3390/diagnostics13152479

**Published:** 2023-07-26

**Authors:** Yohan Guichoud, Oumama El Ezzi, Anthony de Buys Roessingh

**Affiliations:** Service de Chirurgie de l’Enfant et de l’Adolescent, Département Femme Mère Enfant, Lausanne University Hospital, 1011 Lausanne, Switzerland

**Keywords:** cleft lip, cleft palate, prenatal ultrasonography, pregnancy, accuracy, detection rate

## Abstract

Precision of cleft lip and/or palate antenatal diagnosis plays a significant role in counselling, neonatal care, surgical strategies and psychological support of the family. This study aims to measure the accuracy of antenatal diagnosis in our institution and the detection rate of cleft lip and/or palate on routine morphologic ultrasonography. In this retrospective observational study, we compared antenatal and postnatal diagnosis of 233 patients followed in our unit. We classified our patients according to the Kernahan and Stark’s classification system: Group 1: facial cleft including labial and labio-maxillary clefts; Group 2: facial cleft including total, subtotal and submucous palatal clefts; Group 3: labio-maxillary-palatal clefts. Out of 233 patients, 104 were antenatally diagnosed with a facial cleft, i.e., an overall detection rate of 44.6%. The diagnosis was confirmed at birth in 65 of these patients, i.e., an overall accuracy of 62.5%. Of the 67 children (29.2%) in Group 1, the screening detection rate was 58.2% with an antenatal diagnostic accuracy of 48.7%. Of the 97 children (41.6%) in Group 2, the screening detection rate was 2% with an antenatal diagnostic accuracy of 50%. Of the 69 children (29.6%) in Group 3, the screening detection rate was 91.3% with an antenatal diagnostic accuracy of 71.4%. Our study demonstrates a relatively poor diagnostic accuracy in prenatal ultrasound, where the diagnosis was inaccurate in one third to one half of patients. It showed great variability in the screening detection rate depending on the diagnostic group observed, as well as a low rate of detection of palatal clefts.

## 1. Introduction

Cleft lip with or without palate (CL ± P) and isolated palatal cleft (CP) are the most frequent maxillofacial anomalies, with an estimated prevalence of 1/1000 living births affected. According to a 2021 meta-analysis, cleft palate (CP), cleft lip (CL), and cleft lip and palate (CLP) global prevalence were reported to be 0.33, 0.3 and 0.45 per 1000 births, respectively [1].

Facial clefts originate from a disorder in the fusion of facial prominences during embryonic facial development. More specifically, primary palatal clefts such as partial cleft lip, complete cleft lip or alveolar cleft develop between the fourth and the seventh week of gestation. The secondary palate is the true palate, consisting of an anterior bony part and a soft posterior part. The secondary palate clefts develop between the sixth and the twelfth weeks of gestation. Many varieties of clefts can occur, depending on the region involved; for example, bifid uvula, cleft of the soft palate, CP or Pierre-Robin Sequence (PRS). Clefts due to primary and secondary palate malformation can be unilateral or bilateral. The classic description presented by Kernahan and Stark [2,3] categorizes CL ± P and CP into three primary groups: Group 1 with a primary palate defect, group 2 with a secondary palate defect and group 3 with a combination of both.

As a result, facial clefts generate esthetical, functional, social and psychological prejudice. Antenatal diagnosis helps to prepare the parents to confront and accept the facial malformation of the baby they are expecting. Diagnostic accuracy has a strong impact on prenatal counselling, explanation of the type of surgical treatments to be expected, as well as follow up and long term sequalae [4,5].

Antenatal 2D ultrasonography (US) with morphology examination between the 20th and the 22nd week of gestation is an important pregnancy screening tool in the detection of malformations. Though the alveolus and palate examination are optional, the mid-sagital profile of the face and the upper lip in the coronal (frontal) view are part of this screening and should be described following to the guidelines of the International Society of Ultrasound in Obstetrics and Gynecology [6]. In Switzerland, this examination is mostly performed by private gynecologists. When CL ± P or CP is suspected at the morphology screening examination, pregnant women are referred to university centers to confirm or define the diagnosis, as well as to evaluate the presence of a possible extension to the secondary palate.

The aim of this study is to measure the accuracy of a diagnosis performed in our university center. As a secondary goal, we shall present data on the detection rate of primary screening done on a regional scale, and on factors such as subtype distribution, gender distribution, associated anomaly and family history.

## 2. Materials and Methods

### 2.1. Population and Ethics

We conducted a retrospective observational study by analyzing the files from our multidisciplinary facial cleft unit database of children born between 1 January 2008 and the 31 December 2018. The approval of the Board of Ethics was a prerequisite for participation. (CER-VD; number 2019-02093). All children born with CL ± P or CP and followed by our interdisciplinary team were included in this study. The exclusion criteria applied to all craniofacial cleft infants born abroad but currently followed by our institution and all infants with craniofacial clefts other than CL ± P or CP.

### 2.2. Data Collection

Data was systematically collected from patients’ and mothers’ files. Clinical data was recorded using the software REDCap (Research Electronic Data Capture) and stored [7] with the approval of Swissethics. The data collected includes: type of the suspected cleft before birth and its classification, gestational age at birth, type of the cleft with its classification at birth as final diagnosis. REDCap programming allowed an automatic inclusion of an item of antenatal and postnatal matching to avoid any bias. In addition, we collected other data on gender, associated anomaly, family history, mother’s age and health conditions, as well as type of antenatal diagnostic error.

For the analysis, we used the classification system of Kernahan and Stark [1,2] and its modifications to describe three subtypes: Group 1 including labial and labio-maxillary (unilateral or bilateral) facial clefts, Group 2 including total, subtotal and submucous facial clefts and Group 3 including variations of labio-maxillary-palatal facial clefts. We subdivided the third group into two sub-groups: unilateral defects and bilateral defects. Minor labial defects were defined as muscular clefts (“frustre” forms). Minor palatal defects, for instance V-shaped hard palate defects, were defined as incomplete secondary palatal defects. Velar defects were defined as soft palate defects without hard palate involvement.

Descriptions of screening examinations were not always available. The locations of these screenings, which were in part performed in primary, secondary and tertiary centers in unknown proportions, are not mentioned. All the data collected from completed antenatal diagnoses comes from reports from our Center.

### 2.3. Outcomes

The primary outcome is the rate of diagnostic matches between antenatal and postnatal diagnosis, which has been termed diagnostic accuracy. The secondary outcome is the measurement of the detection rate of the primary screening. In order to reduce bias, ultrasonographers’ reports where a subjective interpretation was made without ultrasound evidence of clefts were considered as negative.

### 2.4. Statistics

In order to measure their accuracy, we used data from patients with antenatally diagnosed CL ± P or CP and compared them with the postnatal final diagnosis. The rate of matches reflects the accuracy of our diagnosis and the performance of our Center. The detection rate of the screening is based on the proportion of positive antenatal ultrasound screening among all patients included in our study.

The statistical analysis is purely descriptive, with qualitative results presented as percentages and quantitative results presented as means with a standard deviation description.

## 3. Results

### 3.1. Population

Of the 269 patients initially selected for our study, 36 were excluded: 22 were born outside of Switzerland, 10 showed postnatally suspected clefts without specialized confirmation, and 4 had other orofacial clefts. A total of 233 patients were thus eligible for analysis.

The M/F gender distribution ratio was of 1.92 for patients with primary palatal defect, 0.79 with secondary palatal defect and 2.43 with mixed primary and secondary palatal defect.

The gestational age at birth was at term in the great majority of cases, with a mean age of 38.7 weeks ± 1.8 SD. A total of 20 (8.6%) were preterm births (<37 weeks), of which three (1.3%) were moderate preterms (36–34 weeks) and 1 (0.4%) was very preterm (>32 weeks). The mean in Group 1 (labial and labio-maxillary (unilateral or bilateral) facial clefts) was 39 weeks ± 1.7 SD with four (6%) preterm and one (1.5%) moderate preterm. In Group 2 (total, subtotal and submucous facial clefts) the mean was 38.5 weeks ± 1.7SD with nine preterms (9.3%) including two (2.1%) moderate preterms and one (1%) very preterm. The mean in group 3 (variations of labio-maxillary-palatal facial clefts) was 38.6 weeks ± 1.5 SD with three (4.4%) near terms.

### 3.2. Diagnosis Accuracy and Screening Ultrasonography Detection Rate

Of the 233 children followed in our unit, 104 had been antenatally diagnosed with a cleft, i.e., an overall detection rate of 44.6%. This diagnosis was confirmed at birth in 65 of these patients, i.e., an overall accuracy of 62.5%. Table 1 summarizes the primary outcome.

In group 1, a majority of patients, 59 (25.3%), had a unilateral defect; 13 of those a minor labial defect.

In group 2, 56 (24%) patients had a hard palate defect; 13 of those had an incomplete defect, a submucosal cleft, none of which were detected antenatally.

In group 3, 55 patients (23.6%) had a unilateral complete defect, including two (0.9%) patients with minor defects affecting both primary and secondary cleft palate. The first had a submucosal cleft palate and a minor left cleft lip, and the second had a left cleft lip and a cleft palate without defect of the alveus.

In order to analyze the factors that affect diagnostic accuracy, in Table 2, we show the data that summarizes types of errors made in antenatal diagnosis. The majority of the diagnoses were placed in the wrong group because they were missing a palatal prolongation of the cleft or misinterpreting the presence of a palatal cleft. Another frequent error is assignment to the wrong subtype, such as missing an alveolar extension in a patient with labial and alveolar cleft. One patient had a good initial antenatal diagnostic, which was not confirmed in our Center, and two other patients had a contralateral minor cleft lip that was not detected antenatally.

### 3.3. Family History and Associated Anomalies

Out of a total of 215 (92%) files mentioning family history, 37 showed that a relative had a facial cleft: 17 (7.9%) were first degree relatives, and 20 (9.3%) were second degree relatives. We have measured and summarized the number of negative screenings in Table 3.

Associated anomalies and syndromes and their distribution within the different groups are outlined in Table 4 and Table 5. Twelve patients evidenced a combination of various types of anomalies or syndromes. This explains why the number of anomalies and syndromes shown in Table 5 may be higher than the number of patients. A total of 55 (23.6%) patients were affected by associated anomalies, and most anomalies were encountered in Groups 2 and 3 with bilateral clefts. In Group 1, only 5 (7.4%) patients were affected by other anomalies, including 1 with Opitz Syndrome. In Group 2, 30 (30.9%) patients had an associated condition, and 10 (10.3%) of them had a specific syndrome. In the unilateral defect subgroup of Group 3, 14 (25.5%) patients had associated anomalies, with syndromes in 3 of them. In the bilateral defects subgroup of Group 3, 5 (35.7%) patients had an associated defect without any syndrome.

A large variety of syndromes were present, affecting 6.4% of our patients (without PRS). Van der Woude syndrome was the most frequent, found in four patients (1.7%). A total of 51 patients were screened for 22q11 deletion (DiGeorge syndrome/velocardiofacial syndrome), with only two positive results. Three patients were diagnosed with an unspecific chromosomal defect with 8p21 microdeletion, 13q3 duplication and chromosome 2 translocation.

The most frequent type of anomaly, which affected 6.4% of all patients, was cardio-vascular. ENT anomalies affected 4.3% of our patients. A total of 3% of children had skeleton malformations. Another 2.2% of patients had anomalies of the genital organs. Anomalies of the central nervous system affected 2.2% of our patients. A total of 1.7% suffered integument disease, 1.3% were affected by eye anomalies, 0.4% showed urinary tract defects with unilateral renal agenesia and another 0.4% showed an anorectal malformation. Pierre Robin sequence was found in 21.7% of patients in Group 2. Specific anomalies and diseases are outlined in Table 5.

## 4. Discussion

Our large monocentric retrospective study shows the relatively low accuracy of ultrasound in describing prenatal cleft lip with or without cleft palate with an overall accuracy rate of 62.5%. It also demonstrates variable detection rates with 58.2%, 91.3% and 2% for CL, CLP and CP, respectively. Given that prenatal diagnostics, as well as its accuracy, have a strong impact on parental counseling, this paper advocates for the implementation of new screening and diagnostic tools on a regional scale.

In the literature, the accuracy of antenatal diagnosis using 2D ultrasound varies between 70.5% [8] and 80.7% [9]. Studies that have utilized both 2D and 3D ultrasound, without direct comparisons between the two techniques, have reported accuracy rates ranging from 43% to 87% [10,11,12,13]. The accuracy of diagnostics in our center is lower, with 62.5% of prenatal diagnoses matching postnatal diagnoses. This variability can be explained by various factors, including gestational age, ultrasound protocol, operator expertise, maternal obesity and position of the fetus in utero [14]. Additionally, accuracy may also vary depending on the type of cleft being analyzed, with CP being particularly challenging to diagnose. In our study, we were able to identify only one case of CP with PRS due to the detection of micrognathia. In studies that utilized both 2D and 3D ultrasound, diagnostic accuracy of cleft palate ranged from 33% [11] to 75% [13], but these results were also highly associated with the presence of other anomalies diagnosed with ultrasound.

As represented in Table 2, there were a total of 19 cases (8%) where the postnatal defect was more extensive than initially predicted by prenatal diagnosis. There are limited studies that provide a comprehensive description of the specific types of errors occurring. Berggren et al. [10] have reported similar types of errors, and for the purpose of comparison, we found that 45% of cases in this study exhibited a postnatal defect that was more extensive than initially predicted by prenatal diagnosis.

The detection rate of antenatal diagnosis using 2D ultrasound is heterogeneous in the literature, with an overall detection rate of CL ± P or CP ranging from 43% to 87.5% [8,9,15,16,17]. Our study yielded results within the lower range of previously published studies, with an overall detection rate of 44.6%. In terms of specific cleft types, the detection rate of CL ± P (represented by groups 1 and 3 in our study) is relatively high, ranging between 57% [17] and 60% [16]. In contrast, the detection of CP is challenging, with reported detection rates ranging from 0% [17] to 11% [16]. In our study, we present a higher detection rate for CL ± P (Groups 1 and 3) in 75% and a comparable detection rate for CP in 2% of cases. Unfortunately, the location of the screening US was not systematically available in our patients’ files. Therefore, we cannot know the proportion of primary diagnostics on screening morphology examination performed in our university center versus examinations performed by private gynecologists.

The distribution of cleft types in our study (29% CL, 42% CLP, and 29% CP) is consistent with the classical description in the literature, which suggests a distribution of 25% CL, 50% CLP and 25% CP [18,19]. Regarding family history, Offerdal et al. published a positive rate of 17% for patients with facial oral clefts [18], which is consistent with the rate found in our study of 15.9%. Regarding associated anomalies, our study’s findings are consistent with the literature. Overall rates of 18% and 26% have been reported, with distribution rates of 5–21% and 39%, for CL ± P and CP, respectively [16,17]. In our study, the overall prevalence of associated anomalies was 23.6%, with a distribution of 17.6% and 30% for CL ± P and CP, respectively.

In their systematic review, Maars et al. reported the proportion of postnatal associated anomalies to be 12.1%, 25.1% and 45.9% for CL, CLP and CP, respectively, with a higher rate in bilateral defects, which is consistent with our findings [20]. The syndrome most frequently associated with CL ± P is the van der Woude Syndrome, accounting for 2% of all cases, which is also in line with our results [21]. In our population, syndromes are more frequent in patients with CP, and this finding is consistent with the existing literature [20]. However, the proportions of anomalies that were antenatally detected and the proportion of cases resulting in abortion were not reported due to the design of our study.

Our study possesses several strengths, one of which is the size of our cohort, which is one of the largest available for a monocentric study. However, our study also has its limitations. First, it is a retrospective study. This limitation presents challenges in analyzing and comparing data of interest, such as the involvement of specific ultrasonographers, the specific ultrasound techniques utilized and whether or not 3D ultrasound was employed. Second, the recruitment method used in this study constrained the data collection process, thereby limiting the measurement of the prevalence of orofacial clefts or the impact of several maternal risk factors. Because cleft lip and palate are frequently associated with genetic syndromes, and the number of medical terminations of pregnancy is not known, it is likely that the reported numbers underestimate the true prevalence of orofacial cleft, distribution of the subtypes, and their associations with other anomalies.

Poor accuracy of the antenatal US diagnostic significantly impacts parents counselling on patient prognosis and planning of short- and long-term treatments. In Group 1, more than half of our patients were misdiagnosed, missing a contralateral defect or an alveola extension of the defect. Five patients with a palatal defect were improperly assigned to Group 3 (see Table 3). In Group 3, one third (28.6%) of the patients were misdiagnosed by missing a contralateral defect or a palatal extension of the defect.

In a recent systematic review and meta-analysis investigating the accuracy of prenatal US to detect cleft palate, Lai et al. demonstrated an overall accuracy combining 2D and 3D of 87% [12]. The 3D US seems to have a positive impact on the accuracy of the antenatal diagnostic of clefts [22,23].

Furthermore, specific US markers, such as the description of the two pterygoid processes [24] or the evaluation of the retronasal triangle [25,26,27], have been described in order to enhance the detection rate and the accuracy of cleft palate. Kathleen and Chueh have recently reviewed multiple first, second and third trimester 2D ultrasonographic markers for CL ± P or CP, including the maxillary gap, frontal space, maxilla-nasion-mandible angle, retronasal triangle, palatino-maxillary diameter and equal sign [28]. As an interesting new tool, the assessment of the alveolar cleft size (≥4 mm) on 2D US has recently been investigated as a predictive factor for secondary cleft palate [29]. However, the retronasal triangle seems to show a greater sensitivity for the detection of cleft palate [30]. After a rigorous evaluation, these techniques could be proposed for integration into either the standards of screening or the classical specialized evaluation of palates in our center.

As recently reported by Tonni et al., fetal magnetic resonance imaging (MRI) is a potentially useful second-line investigation for the prenatal diagnosis of orofacial malformations, with a pooled sensitivity of 97% [31]. Additionally, new and interesting approaches, such as the evaluation of maternal serum biomarkers [32], are currently being assessed for their potential in detecting palatal clefts.

More simply, implementing a systematic referral strategy to our Center for patients with a positive family history has the potential to significantly increase the detection rate of clefts. Furthermore, Fuchs et al. have published a quality control tool for assessing the hard palate during the first-trimester ultrasound screening [33].

## 5. Conclusions

Although the number of patients supported by our cleft team is important, the number of patients referred to our university hospital for specialized antenatal ultrasound examination is relatively low. This highlights the need to analyze current modalities and methods used for screening on a regional scale. We advocate referring more systematically to tertiary centers in cases of high-risk pregnancies. Our study challenges current methods of antenatal diagnosis of CL ± P and CP. There is a need for prospective studies of these assumptions. This could inspire new publications proposing changes in our methods of screening and diagnosing those frequent facial clefts; thus, the counselling provided to the patients’ families would be improved.

## Figures and Tables

**Table 1:** Clinical form distribution and primary outcome.

		Total (*n* = 233) *n* (%)	Positive Antenatal US *n* (% = Detection Rate)	Positive Match *n* (% = Accuracy)
Group 1	Unilateral CL	32 (13.7%)	12 (37.5%)	8 (66.7%)
	Unilateral CL+A	27 (11.6%)	20 (74.1%)	10 (50%)
	Bilateral CL+A	6 (2.6%)	6 (100%)	1 (50%)
	Bilateral CL	2 (0.9%)	1 (50%)	0 (0%)
	**Total**	67 (29.2%)	39 (58.2%)	19 (48.7%)
Group 2	CP	56 (24.0%)	0 (0%)	0 (0%)
	PRS	21 (9.0%)	2 (9.5%)	1 (50%)
	CV	20 (8.6%)	0 (0%)	0 (0%)
	**Total**	97 (41.6%)	2 (2%)	1 (50%)
Group 3	Unilateral CL+A+P	55 (23.6%)	50 (90.9%)	35 (70%)
	Bilateral CL+A+P	14 (6%)	13 (92.9%)	10 (76.9%)
	**Total**	69 (29.6%)	63 (91.3%)	45 (71.4%)
All		233 (100%)	104 (44.6%)	65 (62.5%)

CL = Cleft lip; CL+A = Cleft lip and alveola; CP = Cleft palate; PRS = Pierre Robin Sequence; CV = Velar cleft; CPCL+A+P = Complete cleft from lip to palate.

**Table 2:** Error type.

		Group 1	Group 2	Group 3	Total
Wrong group	From 3 to 1	5	-	-	5
	Retrognathism without cleft	-	2	-	2
	From 1 to 3	-	-	11	11
Wrong subtype		11	0	0	11
Bilateral vs. Unilateral		3	-	4	7
Wrong side		1	-	0	1
Other		1 *	0	2 †	3
Total		20	2	17	

* Good diagnostic in primary screening not confirmed in our center. † Contralateral minor labial cleft not detected.

**Table 3:** Family history of clefts and impact on screening.

	Total (*n* = 233) *n* (%)	Negative Screening (*n* = Total) *n* (%)
First degree	17 (7.3%)	8 (47%)
Second degree	20 (8.6%)	5 (25%)
Unknown	18 (7.7%)	16 (88%)
Negative	178 (76.4%)	99 (55.6%)

**Table 4:** Other child anomalies and associated syndromes.

		Group 1 (*n* = 67) *n* (%)	Group 2 (*n* = 97) *n* (%)	Group 3 (*n* = 69) *n* (%)		Total (*n* = 233) *n* (%)
				Unialteral (*n* = 55)	Bilateral (*n* = 14)	
Total patients with associated anomalies or syndromes *		5 (7.4%)	30 (30.9%)	14 (25.5%)	5 (35.7%)	55 (23.6%)
Associated anomalies	CV	3 (4.5%)	6 (6.2%)	4 (7.3%)	2 (14.3%)	15 (6.4%)
	ENT	0	9 (9.3%)	0	1 (7.1%)	10 (4.3%)
	Skeleton	0	6 (6.2%)	1 (1.8%)	0	7 (3%)
	Genitals	0	1 (1.0%)	2 (3.6%)	2 (14.3%)	5 (2.2%)
	CNS	1 (1.5%)	2 (2.1%)	2 (3.6%)	0	5 (2.2%)
	Integument	0	3 (3.1%)	0	0	4 (1.7%)
	Eyes	0	1 (1.0%)	1 (1.8%)	1 (7.1%)	3 (1.3%)
	Urinary tract	0	1 (1.0%)	0	0	1 (0.4%)
	GI	0	1 (1.0%)	0	0	1 (0.4%)
Syndromes	Van der Woude	0	2	2	0	4
	DiGeorge	0	2	0	0	2
	Down	0	1	0	0	1
	Kabuki	0	1	0	0	1
	Treacher-Collins	0	1	0	0	1
	Smith-Lemli-Opitz-	0	1	0	0	1
	Apert	0	1	0	0	1
	Opitz	1	0	0	0	1
	Stickler	0	1	0	0	1
	Wolf-Hirschhorn-	0	1	0	0	1
	West	0	1	0	0	1
	Pitt-Hopkins	0	0	1	0	1
	Total syndromes *	1 (1.5%)	10 (10.3%)	3 (5.5%)	0	14 (6%)
Other chromosomic defects		0	2 (2.1%)	1 (1.8%)	0	3 (1.3%)

CV = Cardio-vascular; ENT = Ear nose and throat; CNS = Central nervous system; GI = Gastro-intestinal. * Does not correlate with total of anomalies or syndromes.

**Table 5:** Detailed characterization of specific anomalies.

CV	Foramen oval	Atrial septal defect	Transposition of great vessels	Epstein malformation
	Ventricular septal defects	Tricuspid atresia	Pulmonary arteries stenosis	Dilated cardiomyopathy
ENT	Retrognathism	Lingual and rhinopharyngeal hamartoma	Ear dysplasia *	
	Sensorineural hearing loss *	low-set ears *	Laryngomalacia	
Skeleton	Clubfoot	Vertebral bodies fusion	Phalanx duplication	
	11 pairs of ribs	Sacral agenesis		
Integument	Hemangioma	Preauricular fistula		
Genitals	Cryptorchidy	Inguinal hernia		
	Micropenis			
CNS	Developmental delay *	Microgyria		
	Hemiparesis	Cerebral pseudocysts	Microcephalus	
Eyes	Blepharophimosis	Iris and retina coloboma	Strabismus	
	Microphthalmia		Pterygium *	
Urinary tract	Unilateral renal agenesia			

* Isolated (without genetic anomaly).

## Data Availability

The data presented in this study are available on request from the corresponding author. However, they are not publicly available as they are encoded and stored on the REDCap program to ensure data security and facilitate data management for future research purposes.
